# Supplementary material for: Five Novel Polymorphs of Cardarine/GW501516 and Their Characterization by X-ray Diffraction, Computational Methods, Thermal Analysis and a Pharmaceutical Perspective
Source: Pharmaceutics. 2024 May 7;16(5):623. doi: 10.3390/pharmaceutics16050623 (PMC11125426; doi:10.3390/pharmaceutics16050623)
Supplement: Supplementary file 1 [file pharmaceutics-16-00623-s001.zip › pharmaceutics-2914085-supplementary.pdf]

Supporting information

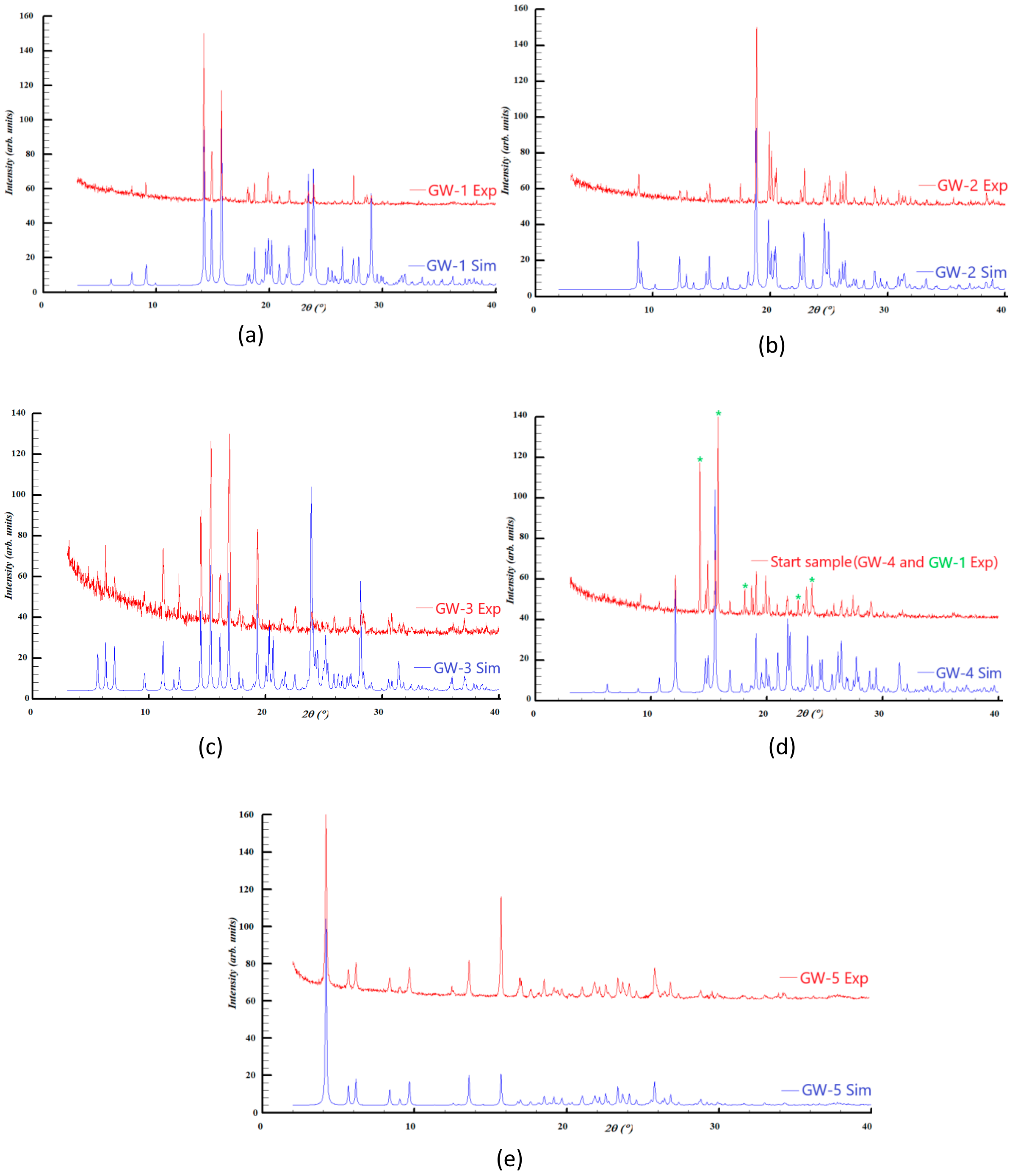

Figure S1. Experimental (Exp) and simulated (Sim) X-Ray diffraction patterns comparison for studied polymorphs: GW-1 (a), GW-2 (b), GW-3 (c), GW-4 (d), GW-5 (e)

Table S1. *Nature and magnitudes of intermolecular interaction energies for selected intermolecular contacts (kJ/mol)*

Crystal	Interaction pair	Contact	E_{ele}	E_{pol}	E_{disp}	E_{rep}	E_{tot}
**GW-1**		O3-H3···N1<br>C11-H11···O3<br>C6-H16···O2	-58.2	-13.4	-21.0	0	-92.6
		C20-H20B···O3<br>C20-H20A···C14 (C-H···π)<br>C20-H20A···C15 (C-H···π)	-18.0	-2.4	-86.7	34.0	-73.1
		C4-H4···O2	-23.4	-4.5	-28.0	21.1	-34.8
		C3-H3···H11A-C11	-3.7	-0.5	-13.8	4.9	-13.1
		C14-H14···S2	-8.0	-2.1	-30.3	14.7	-25.5
**GW-2**		O3-H3···N1<br>C6-H6...O3	-67.5	-12.3	-30.0	55.5	-54.3
		C4-H4···O3<br>C14-H14···C3<br>C15-H15···F2	-13.4	-1.6	-46.1	20.1	-41.0
		C12-H12B···O2<br>C11-H11B···O2<br>C20-H20A···C14 (C-H···π)	-26.1	-3.2	-31.4	0	-60.7
		C11-H11A···F3	-5.8	-0.7	-18.8	0	-25.3
		C19-H19A···H11C-C11	-0.5	-0.2	-7.0	2.8	-4.9
**GW-3**		O3-H3···N1<br>O3-H3···C10	-46.9	-12.6	-18.7	0	-77.6
		C12-H12B···S2<br>C20-H20A···C16 (C-H···π)	-17.6	-2.5	-88.7	35.9	-72.9
		C6-H6···O3	-3.5	-0.6	-13.1	0	-17.2
		C20-H20B···O2	-12.4	-2.2	-7.1	0	-21.7
**GW-4**	Molecule A-Molecule B (asymmetric unit)	O3A-H3A···O2B<br>O3B-H3B···O2A	-114.6	-23.7	-9.8	0	-148.1
	Molecule A-Molecule A	C20A-H20A···C14A (C-H···π)<br>C20A-H20A···C15A (C-H···π)<br>C11A-H11C···C8A (C-H···π)	-19.7	-3.2	-86.6	34.6	-74.9
	Molecule A-Molecule B	C21B···O3A<br>C21A···O3B	10.7	-2.1	-15.3	0	-6.7
	Molecule A-Molecule B	S1A ···F3B	-1.3	-0.4	-11.1	0	-12.8
	Molecule A-Molecule B	C11A-H11B···H11F-C11B<br>C11A-H11B···H14B-C14B	-5.5	-1.3	-25.0	9.1	-22.7
	Molecule A-Molecule B	C12A-H12A···C2B	-2.1	-0.5	-18.5	53.	-15.8
	Molecule A-Molecule B	C7B-H7B···O2A	-10.7	-2.4	-14.1	8.3	-18.9
	Molecule A-Molecule B	F1A ···S1B	-2.3	-0.5	-18.5	6.8	-14.5
	Molecule A-Molecule B	C12B-H12B···F3A	-4.3	-0.4	-13.3	0	-18.0
	Molecule A-Molecule B	C7A-H7A···O2B	-9.9	-2.2	-13.9	7.3	-18.7
	Molecule B-Molecule B	C20B-H20C···O2B<br>C20B-H20D···C14B (C-H···π)<br>C20B-H20D···C15B (C-H···π)	-19.3	-2.7	-86.2	35.5	-72.7
	Molecule B-Molecule B	C19B-H19F···F1B	-2.3	-0.1	-4.0	0	-6.4
**GW-5**	Molecule A-Molecule A	O3A-H3A···O2A	-106.4	-23.5	-10.9	0	-140.8
	Molecule A-Molecule A	O20A-H20D···C15A (C-H···π)<br>O20A-H20D···C14A (C-H···π)<br>C19A-H19F···O2A	-9.5	-3.2	-81.4	25.8	-68.3
	Molecule A-Molecule B (asymmetric unit)	O3B-H3B···N1A<br>C6A-H6A···O2B<br>C11A-H11F···O3B	-47.6	-13.5	-27.5	0	-88.6
	Molecule A-Molecule B	C19B-H19B···O3A	-2.8	-0.7	-10.7	0	-14.2
	Molecule A-Molecule A	C3A-H3AA···F3A	-8.9	-0.6	-9.4	0	-18.9
	Molecule A-Molecule B	C19A-H19E···H20B-C20B	1.1	-1.1	-12.0	0	-12.0
	Molecule A-Molecule B	C6A···O3B<br>C7A···O3B	2.5	-0.6	-14.4	0	-12.5
	Molecule B-Molecule B	C12B-H12B···S1B<br>C12B-H12A···C13B (C-H···π)<br>C12B-H12A···C18B (C-H···π)	-23.0	-4.3	-92.5	37.9	-82.2

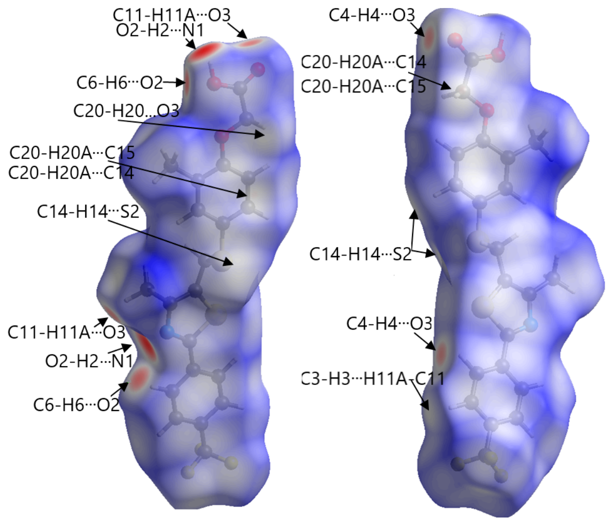

C11-H11A···O3
O2-H2···N1
C6-H6···O2
C20-H20...O3
C20-H20A···C15
C20-H20A···C14
C14-H14···S2
C11-H11A···O3
O2-H2···N1
C6-H6···O2
C4-H4···O3
C20-H20A···C14
C20-H20A···C15
C14-H14···S2
C4-H4···O3
C3-H3···H11A-C11

(a) GW-1

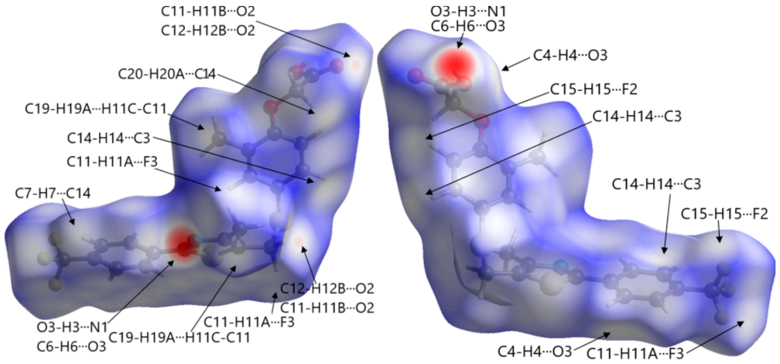

C11-H11B···O2
C12-H12B···O2
C20-H20A···C14
C19-H19A···H11C-C11
C14-H14···C3
C11-H11A···F3
C7-H7···C14
O3-H3···N1
C6-H6···O3
C19-H19A···H11C-C11
C11-H11A···F3
C12-H12B···O2
C11-H11B···O2
O3-H3···N1
C6-H6···O3
C4-H4···O3
C15-H15···F2
C14-H14···C3
C14-H14···C3
C15-H15···F2
C4-H4···O3
C11-H11A···F3

(b) GW-2

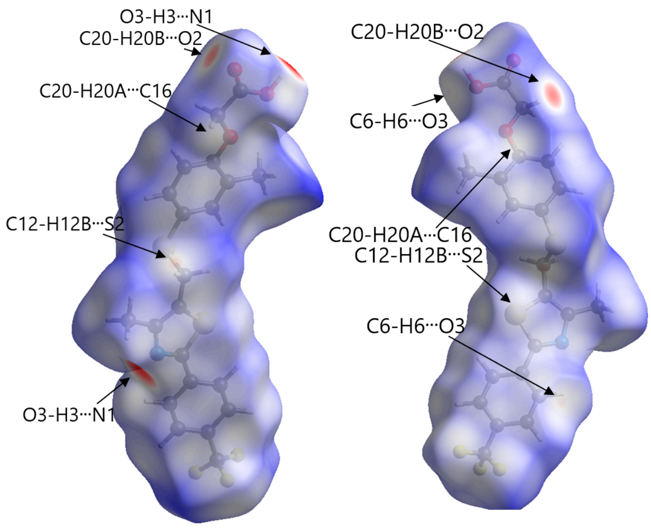
O3-H3···N1
C20-H20B···O2
C20-H20A···C16
C12-H12B···S2
O3-H3···N1
C20-H20B···O2
C6-H6···O3
C20-H20A···C16
C12-H12B···S2
C6-H6···O3

(c) GW-3

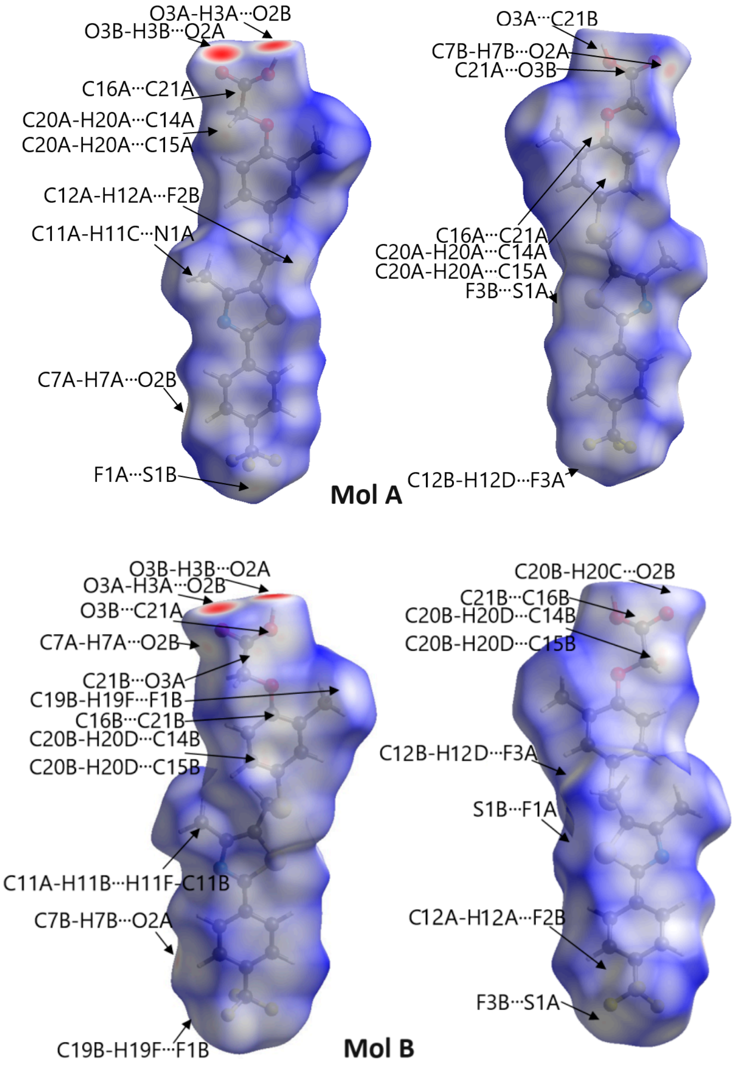
O3A-H3A···O2B
O3B-H3B···O2A
C16A···C21A
C20A-H20A···C14A
C20A-H20A···C15A
C12A-H12A···F2B
C11A-H11C···N1A
C7A-H7A···O2B
F1A···S1B
Mol A
O3A···C21B
C7B-H7B···O2A
C21A···O3B
C16A···C21A
C20A-H20A···C14A
C20A-H20A···C15A
F3B···S1A
C12B-H12D···F3A
O3B-H3B···O2A
O3A-H3A···O2B
O3B···C21A
C7A-H7A···O2B
C21B···O3A
C19B-H19F···F1B
C16B···C21B
C20B-H20D···C14B
C20B-H20D···C15B
C11A-H11B···H11F-C11B
C7B-H7B···O2A
C19B-H19F···F1B
Mol B
C20B-H20C···O2B
C21B···C16B
C20B-H20D···C14B
C20B-H20D···C15B
C12B-H12D···F3A
S1B···F1A
C12A-H12A···F2B
F3B···S1A

(d) GW-4

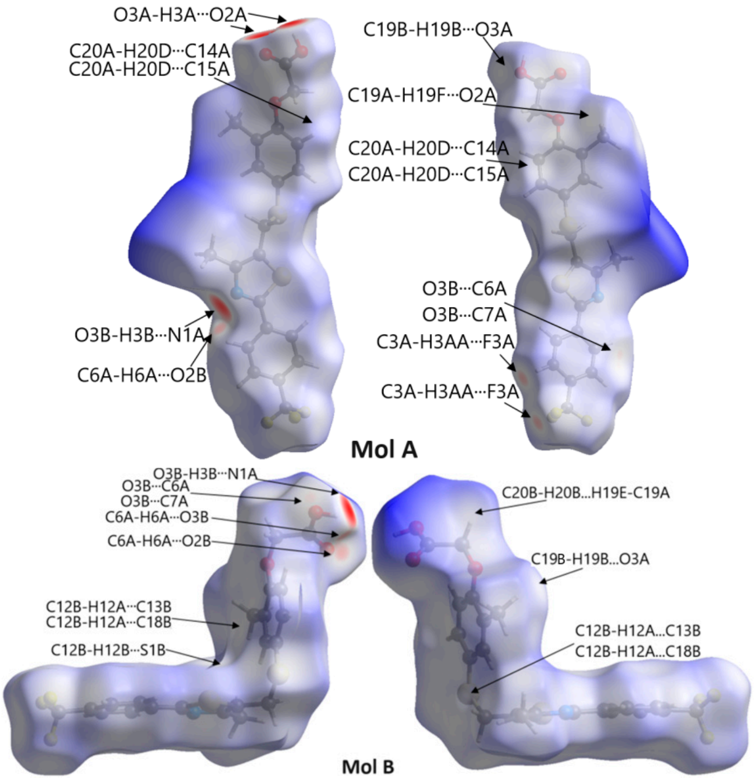

(e) GW-5

Figure S2. Hirshfeld surfaces mapped with dnorm illustrating the contacts referred to in Table S2. Surfaces were represented with the color scale in the ranges as follows: −0.63 (red) to 1.41 (blue) for GW-1 (a); −0.67 (red) to 1.51 (blue) for GW-2 (b), −0.68 (red) to 1.71 (blue) for GW-3 (c), −0.74 (red) to 1.34 (blue) for GW-4 (d), −0.62 (red) to 2.83 (blue) for GW-5 (e)

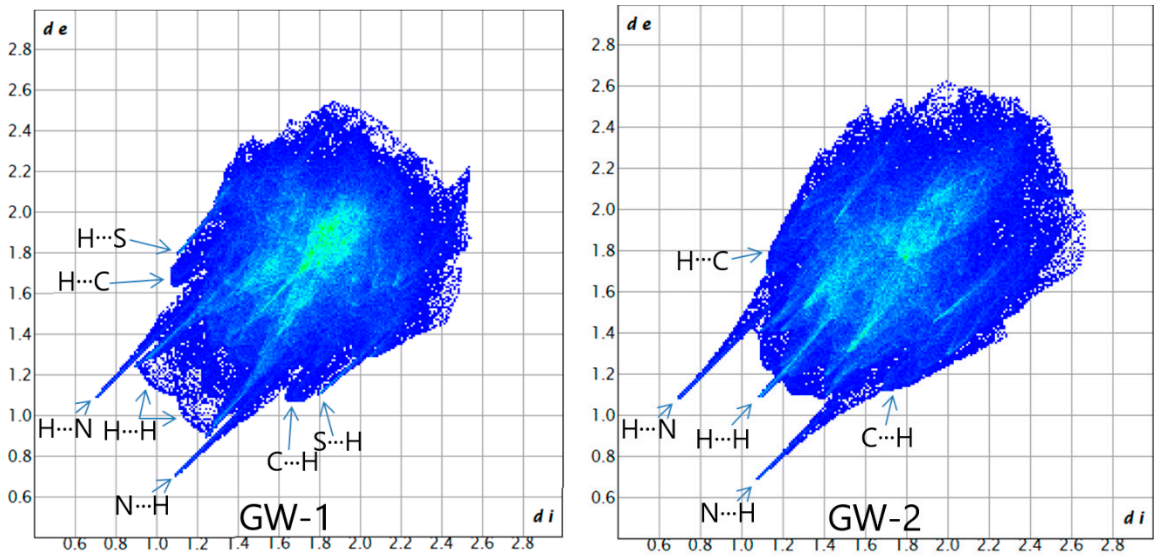

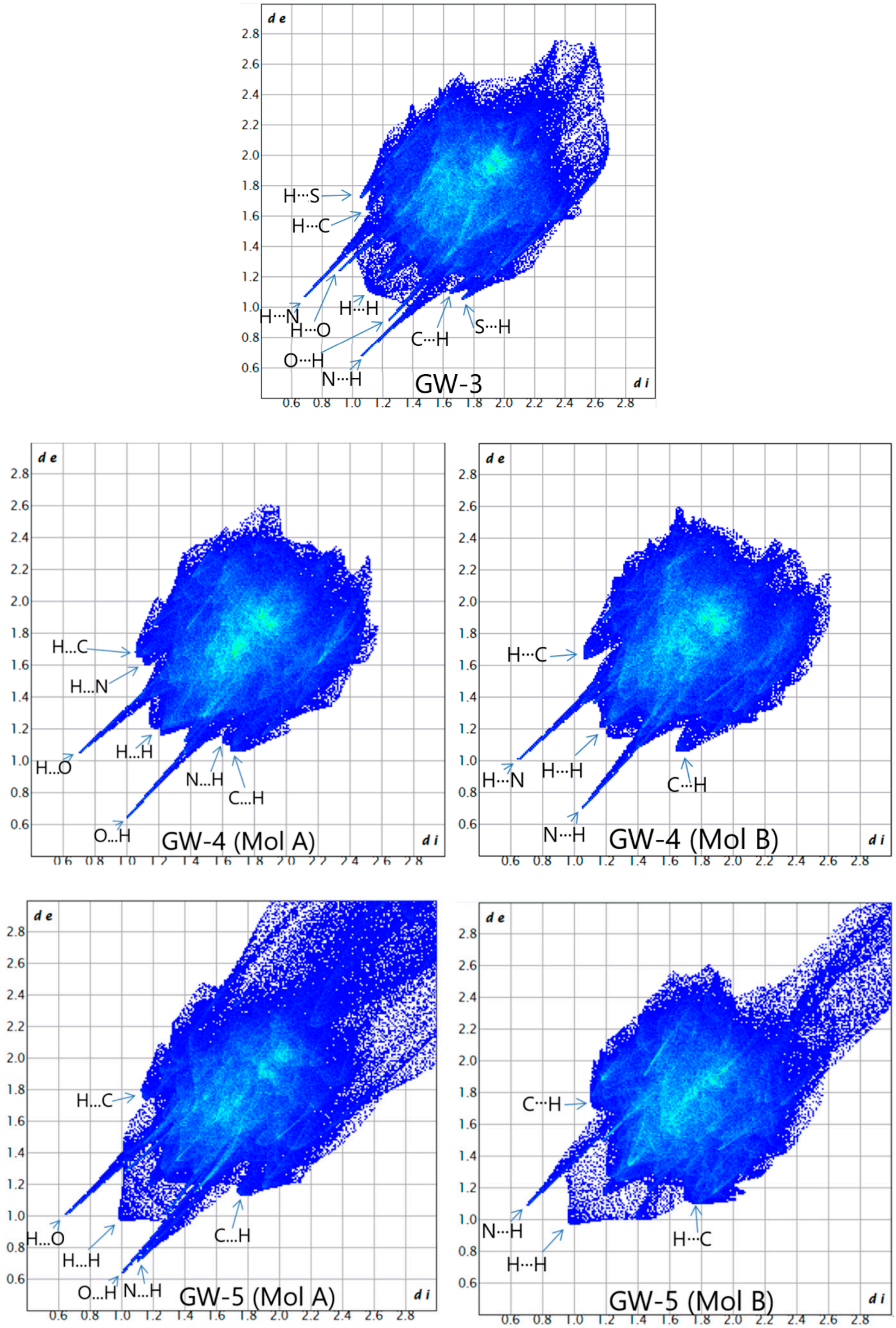

Figure S3. Fingerprint plots of analyzed polymorphs

***Table S2.** Intermediate pairwise interactions for studied polymorphs (Å, °)*

Structure	D-H···A	D-H	H···A	D···A	<(D-H···A)
GW-1	C11-H11A···O3	1.089	2.247(3)	3.255(1)	119.6(4)
	O2-H2···N1	0.993	1.804(2)	2.768(1)	162.7(1)
	O3-H3···N1	1.089	2.158(3)	3.214(1)	162.1(5)
	C20-H20···O3	1.089	2.611(5)	3.434(2)	131.7(4)
	C20-H20A···C15	1.089	2.743(2)	3.640(4)	139.4(4)
	C20-H20A···C14	1.089	2.787(5)	3.777(2)	151.1(1)
	C14-H14···S2	1.089	2.928(2)	4.001(2)	168.3(7)
	C11-H11A···O3	1.089	2.247(2)	3.255(1)	153.1(1)
	C4-H4···O3	1.089	2.267(3)	3.167(2)	138.6(3)
	C3-H3···H11A-C11	1.089		2.372(3)	
GW-2	C11-H11B···O2	1.089	2.604(2)	3.584(2)	149.3(3)
	C12-H11B···O2	1.089	2.502(1)	3.573(3)	167.6(2)
	C20-H20A···C14	1.089	2.763(2)	3.838(2)	169.4(3)
	C21-H21B···H21B-C21	1.089		2.170(1)	
	C14-H14···C3	1.089	2.812(5)	3.891(2)	170.1(1)
	C11-H11A···F3	1.089	2.607(1)	3.379(1)	127.3(3)
	C7-H7···C14	1.089	2.856(2)	3.777(2)	142.4(2)
	O3-H3···N1	0.993	1.759(2)	2.709(1)	159.1(1)
	C6-H6···O3	1.089	2.603(2)	3.392(2)	129.3(1)
	C4-H4···O3	1.089	2.552(4)	3.192(1)	116.6(6)
	C15-H15···F2	1.089	2.604(3)	3.384(2)	127.8(2)
GW-3	O3-H3···N1	0.993	1.743(2)	2.732(1)	173.2(1)
	C20-H20B···O2	1.089	2.162(5)	3.238(3)	169.2(1)
	C20-H20A···C16	1.089	2.741(1)	3.754(4)	154.6(5)
	C12-H12B···S2	1.089	2.784(3)	3.771(1)	150.5(2)
	C6-H6···O3	1.089	2.661(1)	3.166(3)	107.7(1)
	C18-H18B···H2B-C2	1.089		2.231(2)	
GW-4	O3A-H3A···O2B	0.993	1.751(2)	2.617(2)	143.5(6)
	O3B-H3B···O2A	0.993	1.647(3)	2.611(1)	162.4(3)
	C21A···C16A	1.089		3.346(2)	
	C20A-H20A···C14A	1.089	2.721(1)	3.771(2)	162.1(2)
	C20A-H20A···C15A	1.089	2.759(1)	3.673(3)	141.3(5)
	C20B-H20D···C14B	1.089	2.711(3)	3.753(2)	159.9(8)
	C20B-H20D···C15B	1.089	2.743(2)	3.644(4)	139.9(1)
	C12A-H12A···F2B	1.089	2.561(2)	3.506(3)	144.7(1)
	C11A-H11C···N1A	1.089	2.726(2)	3.764(1)	159.3(1)
	C7A-H7A···O2B	1.089	2.475(1)	3.275(3)	129.3(1)
	C12B-H12D···F3A	1.089	2.482(2)	3.411(2)	142.3(8)
	F1A···S1B			3.211(1)	
	F3B···S1A			3.231(2)	
	O3A···C21B			3.171(2)	
	O3B···C21A			3.173(4)	
	C16A···C21A			3.346(3)	
	C16B···C21B			3.361(1)	

	C7B-H7B···O2A	1.089	2.412(1)	3.247(2)	132.3(2)
	C19B-H19F···F1B	1.089	2.641(1)	3.535(2)	138.9(1)
GW-5	O3B-H3B···N1A	1.089	1.813(1)	2.706(1)	148.1(2)
	O3A-H3A···O2A	0.993	1.646(2)	2.627(2)	168.7(2)
	C20A-H20D···O14A	1.089	2.891(4)	3.926(4)	158.8(1)
	C20A-H20D···O15A	1.089	2.887(2)	3.798(5)	141.2(5)
	C6A-H6A···O2B	1.089	2.421(1)	3.483(2)	164.2(2)
	C19B-H19B···O3A	1.089	2.695(3)	3.641(1)	144.9(1)
	C19A-H19F···O2A	1.089	2.601(1)	3.551(2)	145.3(3)
	C3A-H3AA···F3A	1.089	2.291(3)	3.376(2)	174.1(1)
	C12B-H12A···C13B	1.089	2.852(2)	3.505(1)	118.5(3)
	C12B-H12A···C18B	1.089	2.886(2)	3.804(2)	142.1(1)
	C12B-H12B···S1B	1.089	2.915(2)	3.753(2)	133.8(5)
	C20B-H20B···H19E-C19A	1.089		2.259(3)	
	O3B···C6A			3.153(1)	
	O3B···C7A			3.193(5)	

***Table S3**. Contributions to the Hirshfeld diagrams for various contacts*

Structure	H···H	F···H/H···F	O···H/H···O	C···H/H···C	S···H/H···S	S···C/C···S	C···C	Others
GW-1	33.2%	14.6%	11.8%	11.5%	7.6%	4.8%	3.4%	13.1%
GW-2	30.1%	22.0%	14.6%	11.5%	6.9%	4.6%	2.7%	7.6%
GW-3	32.2%	14.4%	13.5%	9.7%	11.3%	1.0%	5.3%	12.6%
GW-4 Mol A	34.2%	16.7%	9.5%	12.6%	8.2%	2.2%	3.0%	13.6%
GW-4 Mol B	33.1%	15.8%	10.2%	12.9%	8.5%	2.3%	3.2%	12.1%
GW-5 Mol A	36.3%	11.3%	14.6%	8.9%	8.0%	2.1%	4.1%	14.7%
GW-5 Mol B	31.9%	8.7%	13.7%	14.2%	10.7%	0.8%	3.2%	16.8%

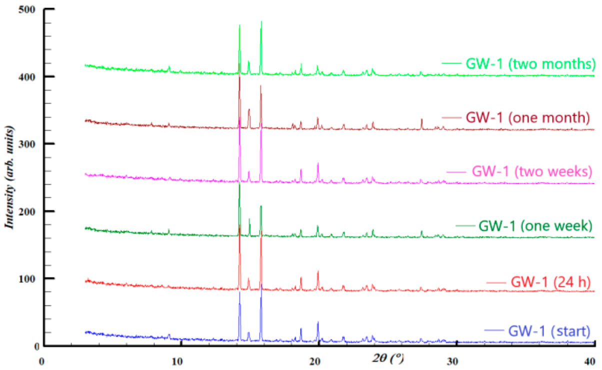

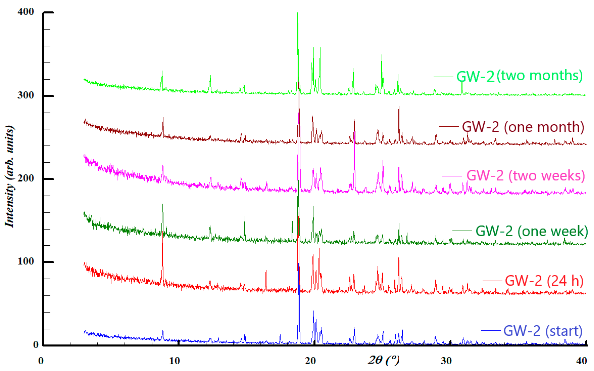

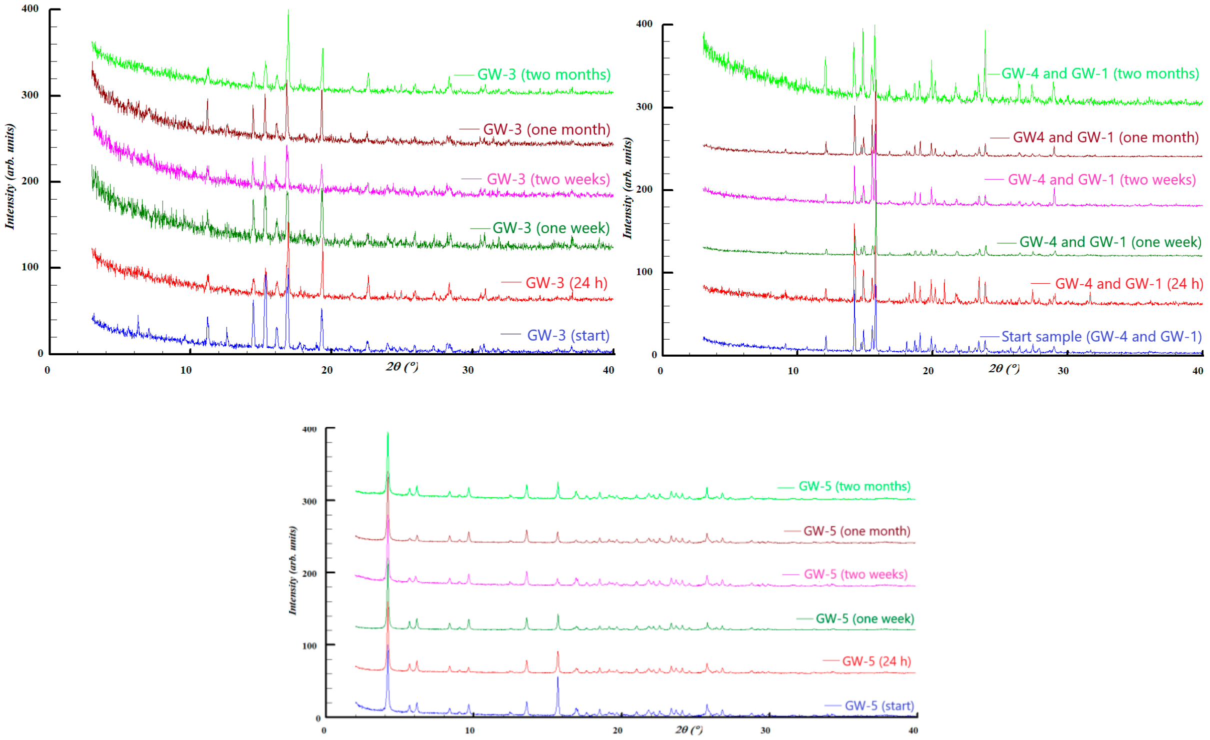

Figure S4. X-Ray diffraction patterns of the polymorphs kept in the climatic chamber
